# A Feasibility Study of a Remotely-Delivered Mindfulness-Based Training for Adolescents During the COVID-19 Pandemic

**DOI:** 10.3389/fpsyt.2022.838694

**Published:** 2022-05-12

**Authors:** Olga Tymofiyeva, Melody Y. Hu, Benjamin S. Sipes, Angela Jakary, David V. Glidden, Namasvi Jariwala, Sarina Bhandari, Kendall C. Parks, Ca Nguyen, Eva Henje, Tony T. Yang

**Affiliations:** ^1^Department of Radiology and Biomedical Imaging, University of California, San Francisco, San Francisco, CA, United States; ^2^Division of Child and Adolescent Psychiatry, Department of Psychiatry and Behavioral Sciences, Langley Porter Psychiatric Institute, University of California, San Francisco, San Francisco, CA, United States; ^3^Department of Epidemiology and Biostatistics, University of California, San Francisco, San Francisco, CA, United States; ^4^Department of Clinical Science, Child and Adolescent Psychiatry, Umeå University, Umeå, Sweden; ^5^Weill Institute for Neurosciences, University of California, San Francisco, San Francisco, CA, United States

**Keywords:** adolescent, mental health, mindfulness, tele-behavioral health, remote

## Abstract

Social distancing, home confinement, economic challenges, and COVID-19-related illness and deaths during the COVID-19 pandemic can significantly affect mental health in youth. One promising approach to reduce anxiety and depression in adolescents is the neuroscience-based mindfulness intervention Training for Awareness, Resilience, and Action (TARA). The objective of this individually randomized waitlist-controlled trial (RCT) was (1) to test the feasibility of TARA, delivered partially over Zoom, and (2) to assess changes in the emotional wellbeing in healthy adolescents between the ages of 14–18 years old during the COVID-19 pandemic. Methods: Twenty-one healthy adolescents were randomized to the TARA intervention or to the waitlist control group in February 2020, just before the start of the pandemic. The TARA group intervention was delivered in person for the first five sessions and remotely over Zoom for the remaining seven sessions due to the pandemic. The participants’ acceptability of TARA was assessed weekly using the Child Session Rating Scale (CSRS). The primary outcome was the emotional wellbeing measured using emotional symptoms subscale of the Strengths and Difficulties Questionnaire (SDQ) pre/post-TARA. We also explored weekly changes in TARA participants’ wellbeing using the Child Outcome Rating Scale (CORS). Results: The overall session rating in TARA participants improved after the switch to Zoom (Cohen’s d = 1.2, *p* = 0.008). The results of the two-way ANOVA showed no statistically significant difference in the change of the SDQ emotional symptoms during the 12 weeks between the TARA group and waitlist-control group (timepoint × group interaction: *F* = 0.77, *p* = 0.38). The exploratory analysis using the CORS in the TARA participants showed a significant improvement in their functioning over the weeks of training. Conclusion: Our results support the feasibility of TARA delivered over Zoom. While our primary outcome did not provide support for the improvement of the emotional wellbeing with TARA compared to a passive control group, our exploratory analysis in the intervention group indicated an improved functioning over the weeks of TARA training. The important general positive impact of this study lies in the possibility of offering a neuroscience-based mindfulness intervention remotely to youth living in remote areas and for all youth during pandemic times.

## Introduction

The COVID-19 pandemic has had an unprecedented impact on the lives of people in the US and across the globe. In addition to the direct medical burden of increasing cases and mortality, COVID-related changes such as shelter-in-place and social distancing also have wide-ranging socioeconomic consequences, all of which are likely contributing to increased incidence of mental health conditions such as depression and/or anxiety. A Centers for Disease Control and Prevention (CDC) cross-sectional study of American adults between April and September 2020 estimated the prevalence of depressive or anxious symptoms at 35–40% (1, 2), representing an approximate 3–4-fold increase compared to that estimated in June 2019 (3).

Adolescents may be even more vulnerable to the negative mental health impacts of the COVID-19 pandemic. Youth already show heightened vulnerability to development of depression and anxiety, with an estimated 62.5% of individuals with onset of any mental disorders happening before the age of 25 years (4). Adolescence is a period of unique developmental sensitivity to social interactions (5), and thus may be particularly vulnerable to adverse effects of COVID-associated social isolation such as shelter-in-place and prolonged school closures. Children and adolescents are also secondarily exposed to stressors on caregivers and family units, such as increased financial insecurity, food insecurity, increased caregiving burden in the setting of reduced childcare options, and new challenges related to confinement conditions (6). Furthermore, COVID-19 may also have impacts on other risk factors for mental health such as adequate sleep (7). Lastly, the impacts of COVID-19 may also limit access to sources of mental health care, given that approximately 1/3 of adolescents receive mental health care in a school setting (8).

Indeed, a recent meta-analysis of 29 studies including 80,879 youth globally showed that the prevalence of depression and anxiety symptoms during COVID-19 has doubled, compared with pre-pandemic estimates, and moderator analyses revealed that prevalence rates were higher when collected later in the pandemic, in older adolescents, and in girls (9). Another longitudinal nationally representative cohort study conducted in China showed that even after the initial control of the COVID-19 (when new COVID-19 cases steadily declined), psychological distress in youth persisted for nearly a half of those participants who reported psychological distress during the initial outbreak of the pandemic (45.04% during the initial outbreak and 26.49% after the control) (10).

A recent systematic review aimed to identify interventions targeting children and their caregivers to reduce psychosocial problems in the course of the COVID-19 pandemic and comparable outbreaks (11). They identified 11 study protocols reporting on trials planned in China, the US, Canada, the United Kingdom, and Hungary during the COVID-19 pandemic (including our trial reported in this paper). Four interventions targeted children ≥ 10 years directly, and seven system-based interventions targeted the parents and caregivers of younger children and adolescents. Outcome measures encompassed mainly anxiety and depressive symptoms, different dimensions of stress or psychosocial wellbeing, and quality of supportive relationships. The authors concluded that there is a paucity of studies on psychosocial interventions for children during the COVID-19 pandemic and further research should be encouraged in light of the expected demand for child mental health care and management (11). The authors also reported that no completed studies on the subject could be identified (11); thus, this paper would be the first to report the results on this subject.

Our current study focuses on a promising approach to reduce anxiety and depression in youth: a neuroscience-based mindfulness intervention Training for Awareness, Resilience, and Action (TARA) that was initially conceptualized and designed to treat adolescent depression (12). The theoretical framework of TARA is aligned with the Research Domain Criteria (RDoC) of the National Institute of Mental Health (NIMH) (13). Specifically, this semi-manualized 12-week group training, informed by mindfulness-, yoga-based techniques and modern psychotherapeutic approaches has been designed to target the neurocircuitry involved in emotional hyper-reactivity, agitation, and dysphoric mood as described in our conceptual paper on TARA (12). Our preliminary findings showed significant improvement in depression, anxiety, and sleep in depressed adolescents following our TARA intervention (14). We also observed an improvement in emotional wellbeing in healthy adolescents with TARA. Specifically, a significant decrease in anxiety symptoms with TARA was observed in healthy adolescents, compared to a control time period (15), as well as changes in structural brain connectivity (15) and gray matter volume (16). TARA is usually delivered over 12 weeks by two facilitators in groups of 10–15 adolescents in-person. Whether the TARA intervention can be delivered remotely has not been previously tested. Other types of therapy delivered remotely, such as internet-based cognitive behavioral therapy, have shown comparable efficiency to face-to-face delivery (17).

The objective of this study was (1) to assess the feasibility of Zoom-delivered TARA, and (2) to utilize an individually randomized group treatment, open-label, waitlist-controlled clinical trial to estimate the effect of TARA (delivered partially over Zoom) in improving the emotional wellbeing (or reducing its worsening) in healthy female and male adolescents between the ages of 14–18 years old during the COVID-19 pandemic. Our hypothesis was that emotional symptoms in adolescents in the intervention group would improve more (or show less deterioration) than in the waitlist control group.

## Materials and Methods

The study was approved by the Institutional Review Board (IRB) of the University of California, San Francisco. All participants in the study provided written informed assent and their parent(s) or legal guardian(s) provided written informed consent in accordance with the Declaration of Helsinki. This study was registered on ClinicalTrials.gov (NCT04548544) prior to data analysis.

The study design was a randomized-controlled trial (RCT) with waitlist controls. More specifically, the study design can be classified as an individually randomized group treatment, open-label, waitlist-controlled clinical trial.

Prior to the pandemic, the study was planned to test TARA’s neural mechanisms (based on MRI) and effects on the wellbeing of youth. The objective was modified retrospectively to include the context of the COVID pandemic and virtual administration of the intervention. The study type was changed from main study to feasibility study, and the sample size was not based on a power analysis. Feasibility studies do not require a prior power analysis or hypothesis testing (18). While we do perform hypothesis testing for the wellbeing outcomes, we report the results cautiously. Because the post-intervention MRIs could not be performed due to the pandemic, the neural mechanisms could not be assessed.

Our inclusion criteria were: Healthy female and male adolescents; 14–18 years old; fluency in English. Our exclusion criteria were: MRI contraindications and pregnancy; current mindfulness training and/or practice with a typically sitting meditation or yoga of 20 or more minutes two or more times per week within 60 days prior to study entry. Adolescent participants were recruited using IRB-approved flyers posted in San Francisco high schools, in neighboring areas, and online.

Twenty-one healthy female and male adolescents (age 15.9 + −1.2 years, 11F/9M/1other) were randomized to the TARA intervention or to the waitlist control group in February 2020, before the start of the before the start of shelter-in-place due to the COVID-19 pandemic. The TARA group intervention was delivered in person for the first five sessions and remotely over Zoom for the remaining seven sessions.

### Feasibility of Training for Awareness, Resilience, and Action Over Zoom

To achieve the 1st objective of this study, to assess the feasibility of Zoom-delivered TARA, the participants’ acceptability of TARA was assessed weekly using the Child Session Rating Scale (CSRS) (19). The CSRS is a 4-item self-assessment using a 10-cm visual analog scale, with higher scores indicating better experience. Participants rated each session in terms of how much they felt listened to (choosing on a continuous scale between “The teachers did not always listen to me.” and “The teachers listened to me.”), how important the content and activities were to them (choosing on a continuous scale between “What we did and talked about was not really that important to me.” and “What we did and talked about were important to me.”), how much they liked the session (choosing on a continuous scale between “I did not like what we did today.” and “I liked what we did today.”), and their overall experience (choosing on a continuous scale between “I wish we could do something different.” and “I hope we do the same kind of things next time.”). We also conducted a Focus Group with the participants in the intervention group.

Solicited adverse events in TARA participants were captured in the following manner: (1) before each TARA session participants were asked through a questionnaire: “Has there been any significant unfavorable change in your mental or physical health since last class?”; (2) if the participant answered “no” to this question, then no additional information was requested; (3) if the participant answers “yes” to this question, then the research assistant or another study staff member asked the participant for additional details regarding the participant’s answer and this information was recorded in the Adverse Event form.

### The Effects of Training for Awareness, Resilience, and Action on Emotional Wellbeing

To estimate the effect of TARA (delivered partially over Zoom) in improving the emotional wellbeing (or reducing its worsening) in healthy adolescents during the COVID-19 pandemic, which was the second objective of this study, the emotional wellbeing primary outcome was measured using the emotional symptoms subscale of the Strengths and Difficulties Questionnaire (SDQ) pre/post-TARA (20). The SDQ was selected because it is widely used in adolescents to measure mental health and has good psychometric properties (21). It has been demonstrated that youth with higher SDQ scores have greater psychopathology as judged by the prevalence of clinical disorder (including emotional disorders such as depression and generalized anxiety disorder), both cross-sectionally and in predicting disorder status 3 years later (20). The emotional symptoms subscale of SDQ includes statements such as “I worry a lot,” “I am often unhappy, depressed or tearful,” etc. Subjects are asked to give their answers on the basis of how things have been for them over the last 6 months. To increase reliability, the SDQ questionnaire was (1) programmed and automatically scored in Qualtrics, and (2) administered electronically to the participants by the same researcher in both the intervention and the control group. No researchers were present in the room when participants filled out the SDQ questionnaire.

Our hypothesis was that emotional symptoms measured using SDQ in adolescents in the intervention group would improve more (or show less deterioration) than in the control group. Two-way-ANOVA was performed.

We also explored weekly changes in TARA participants’ wellbeing using the Child Outcome Rating Scale (CORS) (19). The CORS is a 4-item self-assessment using a 10-cm visual analog scale, with higher scores indicating better functioning. CORS has been found to be psychometrically robust (22). Specifically, respondents answer questions about themselves (How am I doing?), family (How are things in my family?), school (How are things at school?), and their general sense of wellbeing/distress (How is everything going?). The CORS data was collected only in TARA participants, on a weekly basis before each session. Our exploratory hypothesis was that there would be an improvement in CORS in the intervention group with time (Pearson’s correlation between CORS and session number). We assessed Pearson’s correlation between weeks and the CORS values, and we also analyzed the CORS data using a linear mixed effects model, with time (weeks) as a fixed factor and participants as a random factor. Mixed models are robust to missingness; they can give unbiased results when people to dropout based on their previous CORS values and extrapolate to the full cohort. Restricted cubic splines was used to model the CORS values over time in a flexible way (23).

SPSS Version 26 was used to conduct the statistical analyses.

## Results

The demographics of the study participants are presented in Table 1. Out of the 21 participants, 12 were randomized into the TARA group and 9 were waitlist controls. The gender composition was 6 female, 6 male in the TARA group and 5 female, 3 male, and 1 other in the control group. The average age was 16 years old in both groups. The distribution by race deviated from the Census estimates for San Francisco County, California (24) in that there was a higher representation of youth who indicated “Other or more than one race” and “Asian” compared to the Census estimates and a lower representation of youth who indicate “White/Caucasian” compared to the Census estimates. There was an uneven distribution of the socio-economic status estimated based on the highest level of education of the mother between the intervention and the control groups. In the control group, 100% of the mothers were at least high school graduates, whereas in the TARA group, only 41% of the mothers were at least high school graduates. On average, approximately 60% of US females are expected to be at least high school graduates (25).

**TABLE 1:** Demographic and baseline characteristics of the study participants.

	TARA group	Control group
Number of participants	12	9
Gender (Female/Male/Other)	6/6/0	5/3/1
Age at baseline	16.0 (1.0; 14.9–18.1)	15.7 (1.4; 14.1–18.1)
**Race**		
White/Caucasian African American Asian Native American Pacific Islander Other or more than one	2 (17%) 1 (8%) 6 (50%) 0 (0%) 0 (0%) 3 (25%)	1 (11%) 0 (0%) 6 (67%) 0 (0%) 0 (0%) 2 (22%)
**Ethnicity**		
Hispanic or Latino Not Hispanic or Latino Unknown	1 (8%) 10 (83%) 1 (8%)	0 (0%) 7 (78%) 2 (22%)
**Socio-economic status/the highest level of education of the mother**		
Less than 9th grade Some high school Graduated high school Some college Graduated college Professional/graduate degree Do not know SDQ emotional problems T-score at baseline	0 (0%) 3 (25%) 3 (25%) 0 (0%) 1 (8%) 4 (33%) 1 (8%) 56.9 (10.0; 41.4–70.0)	0 (0%) 0 (0%) 0 (0%) 2 (22%) 3 (33%) 4 (44%) 0 (0%) 57.3 (10.9; 36.7–70.0)

*SDQ,Strengths and Difficulties Questionnaire.*

There were no dropouts from the study and no intervention-related adverse events were reported.

The overall participant acceptability of the TARA interventions was high as measured by the “Overall experience” CSRS subscale: averaging 8.5 out of 10.0 across all of the participants, sessions, and questions (how much participants felt listened to, how important the content and activities were to them, how much they liked the session, and their overall experience). On average, participants attended 8 (67%) of the TARA sessions. Three participants attended fewer than 50% of the sessions.

### Feasibility of Training for Awareness, Resilience, and Action Over Zoom

Paired *t*-test analysis between the initial in-person format and later Zoom format showed significant improvement in the “Overall experience” CSRS subscale (*p* = 0.008) with a large effect size (Cohen’s d = 1.16). The other subscales did not show significant differences between the in-person and Zoom portions of the TARA intervention (“How well I was listened to” *p* = 0.365, “How important” *p* = 0.296, “What we did” *p* = 0.365). The three participants who attended fewer than 50% of the sessions were excluded from this analysis due to dropping out of the study after the switch to Zoom format.

Five out of 12 participants in the TARA group agreed to participate in the Focus Group. The participants’ responses to the questions, “How was your experience switching from in-person classes to Zoom?” and “How did the TARA program in general impact you?” are shown in Supplementary Material 1. Overall, switching to Zoom was associated with more distractions that the participants experienced at home, but one adolescent participant also mentioned the convenience of remote classes compared to in-person. There was a consensus that switching to Zoom was better than not doing TARA at all. In terms of the general impact of TARA, the adolescent participants noted an improved wellbeing and better regulation of emotions. One participant noted it helped them with a sense of purpose and belonging.

### The Effects of Training for Awareness, Resilience, and Action on Emotional Wellbeing

The results of the two-way ANOVA showed no statistically significant difference in the change of the SDQ emotional symptoms subscale (primary outcome) during the 12 weeks between the TARA group and waitlist-control group (timepoint × group interaction: *F* = 0.77, *p* = 0.38). There were no statistically significant changes after the 12 weeks in either group.

The exploratory analysis of CORS data in *n* = 12 participants in the TARA intervention group showed a significant positive correlation between weeks and the mean CORS-me weekly ratings (averaged across all 12 participants; Pearson correlation coefficient *r* = 0.677, *p* = 0.016), as well as for the mean CORS-everything weekly ratings (Pearson correlation coefficient *r* = 0.645, *p* = 0.023).

**FIGURE 1:**
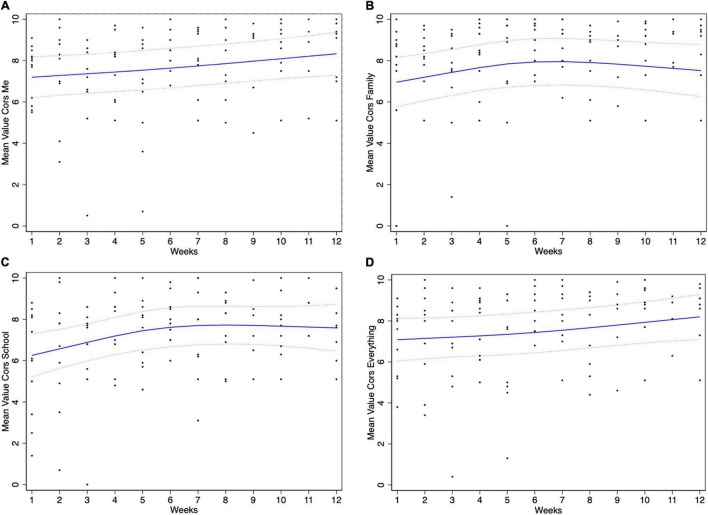
Mean Changes in Child Outcome Rating Scale (CORS) scores in TARA participants based on linear mixed models with restricted cubic splines—dashed linear are estimates plus or minus one standard error. The changes are presented for the four items of CORS self-assessed by the participants and describing **(A)** themselves (How am I doing?), **(B)** family (How are things in my family?), **(C)** school (How are things at school?), and **(D)** their general sense of wellbeing/distress (How is everything going?).

In the analysis of the CORS data using a mixed effects model and restricted cubic splines (Figure 1), with time (weeks) as a fixed factor and participants as a random factor, we found a significant effect over weeks for scores of CORS-me (*p* < 0.000), CORS-school (*p* = 0.009), and CORS-everything (*p* = 0.002). The average score for CORS-me was 1.13 points higher at week 12 compared to week 1. The average score for CORS-everything was 1.12 points higher at week 12 compared to week 1. The average score for CORS-school was 1.33 points higher at week 12 compared to week 1. The average score for CORS-family was 0.56 points higher at week 12 compared to week 1; however, this result was not statistically significant, *p* = 0.17.

## Discussion

Overall, our results support the feasibility of TARA delivered remotely over Zoom. There was even an increase in participant acceptability of the TARA interventions as measured by the “Overall experience” CSRS subscale after switching to Zoom. Results of the Focus Group suggest that switching to Zoom may have been associated with more distractions that the participants experienced at home, but one person also mentioned the convenience of remote classes compared to in-person. There was a consensus among the Focus Group participants that switching to Zoom was better than not doing TARA at all.

Because it is a feasibility study, the results of hypothesis testing for the wellbeing outcomes need to be interpreted cautiously (18). Our primary outcome (SDQ emotional symptoms) did not provide support for the improvement (or reduced worsening) of the emotional wellbeing with TARA compared to a passive control group during the COVID-19 pandemic. There were no statistically significant changes after the 12 weeks in either group, and one possible explanation is that the SDQ refers to the subject’s experience over the last 6 months and might be not sensitive enough to the changes over a 12-week time period. Because of the SDQ referring to the subject’s experience over the last 6 months, it is also more prone to the recall bias compared to scales that refer e.g., to the last week, such as CORS. It is also worth noting that the socio-economic status estimated based on the highest level of education of the mother differed between the intervention and the control groups. In the control group, 100% of the mothers were at least high school graduates, whereas in the TARA group, only 41% of the mothers were at least high school graduates. The impact of the pandemic could have more negatively affected families with a lower socio-economic status. Our exploratory analysis using the CORS in the TARA participants showed a significant improvement in their functioning over the weeks of training for the subscales CORS-me, CORS-school, and CORS-everything (but not CORS-family). In terms of the general impact of TARA, the Focus Group participants noted an improved wellbeing and better regulation of emotions. One participant noted it helped them with a sense of purpose and belonging.

Limitations of the study are the small sample size and the switch from in-person to Zoom delivery of TARA mid-intervention. The distribution by race deviated from the Census estimates for San Francisco County (California), and there was an uneven distribution of the socio-economic status estimated based on the highest level of education of the mother between the intervention and the control groups. Another limitation is the missing data in CSRS and CORS weekly measures due to the variable attendance. To better handle missing CORS data, we used mixed models which allow for adolescent participants to dropout based on their previous CORS values and extrapolate to the full cohort. Restricted cubic splines were used to model the CORS values over time in a flexible way. Although we observed an increase in participant acceptability of the TARA interventions as measured by the “Overall experience” CSRS subscale after switching to Zoom, this observation was after excluding three participants who attended fewer than 50% of the sessions due to dropping out of the study after the switch to Zoom format.

Interventions aimed at reducing depression and anxiety symptoms in adolescents are essential to help prevent development of Major Depressive Disorder (MDD), a highly prevalent illness ranked as the #1 leading cause of disability worldwide (3) that often begins during adolescence. The important general positive impact of this study lies in the possibility of offering a neuroscience-based mindfulness intervention remotely to youth living in urban and remote rural areas and for all youth during both pandemic and non-pandemic times. Such possibility is especially important given the current lack of sufficient access to adequate mental health care for adolescents in both urban and rural areas as well as the potential for future pandemics due to variants of COVID-19 and other viruses which further exacerbate the lack of sufficient access to adequate mental health care for adolescents. Future larger studies are needed to assess efficacy and real-life efficiency of the remotely delivered TARA, in which participants consistently undergo all 12 sessions remotely. Future research would benefit from inclusion of an active control group and from additional efforts to achieve a more representative demographic distribution.

## Data Availability Statement

The raw data supporting the conclusions of this article will be made available by the authors, without undue reservation.

## Ethics Statement

The studies involving human participants were reviewed and approved by Institutional Review Board (IRB) of the University of California San Francisco. Written informed consent to participate in this study was provided by the participants’ parent(s) or legal guardian(s). Written informed assent to participate in this study was provided by the participants.

## Author Contributions

OT, MH, EH, and TY conceived the work. BS, AJ, NJ, SB, and KCP conducted data collection. OT, MH, BS, DG, and CN conducted data analyses. DG prepared the figures. OT and MH drafted the work. OT, MH, BS, AJ, EH, and TY interpreted the data. All authors co-wrote the manuscript.

## Conflict of Interest

The authors declare that the research was conducted in the absence of any commercial or financial relationships that could be construed as a potential conflict of interest.

## Publisher’s Note

All claims expressed in this article are solely those of the authors and do not necessarily represent those of their affiliated organizations, or those of the publisher, the editors and the reviewers. Any product that may be evaluated in this article, or claim that may be made by its manufacturer, is not guaranteed or endorsed by the publisher.
