# Supplementary material for: A Feasibility Study of a Remotely-Delivered Mindfulness-Based Training for Adolescents During the COVID-19 Pandemic
Source: Front Psychiatry. 2022 May 12;13:838694. doi: 10.3389/fpsyt.2022.838694 (PMC9133427; doi:10.3389/fpsyt.2022.838694)
Supplement: Supplementary file 1 [file Table_1.DOCX]

Supplementary Material

**Focus Group**

*Question: “How was your experience switching from in-person classes to Zoom?”*

*Answers:*

Bcxxx: It’s definitely less personal and it’s harder to focus, but it wasn’t terrible. I would rather do it on zoom than stop doing it.

Bcxxx: I got distracted more easily with things on the desk, shelf, dogs barking, but I do agree that it’s better than not doing it at all.

Bcxxx: I agree with J because the environment I was in was different. Before everyone would be focusing on doing the meditation and yoga, but in my house there are all my other family members walking and talking.

Bcxxx: Same for me. But then I feel like I would like doing it at home better because it’s a more comfortable place for me. And, like, it feels more personal. And at the same time I also get distracted. And it’s easier to just, like, stay home, because you don’t have to go to—you don’t have to take a bus and go home late.

Bcxxx: I’m going to pretty much reiterate: it’s pretty distracting for me being near my family and doing things at home. Especially homework. So doing these sessions on zoom was a real challenge for me. And it’s okay with everything going on right now, I’m glad to have this.

*Question: “How did the TARA program in general impact you?”*

*Answers:*

Bcxxx: For behavior and actions I don’t get mad as easily. Or if I do, it’s easier to calm myself down so I don’t lash out and get mad at other people. So I’m better at that now.

Bcxxx: For me it was mostly my wellbeing because it would help with anxiety levels and stress levels. But with emotions and relationships I didn’t see any change.

Bcxxx: For me it impacted my wellbeing definitely and emotions. And then relationships with other people a little bit, and also behavior a lot. So basically it’s just to help me relax. I just think maybe when I’m irritated or frustrated I would like think of the thing I did and it would calm me down.

Bcxxx: For me, I think I want to say I’m pretty much the same except that I picked up, like, new good habits. So that obviously translates to better wellbeing. Habits: yoga and meditation.

Bcxxx: Since I’m a senior in high school doing TARA really helped me with a sense of purpose and belonging because it’s my last year and then like, having anxiety and trying to see that I’m going to be transitioning to college, the practices have helped me. It’s also helped me regulate my emotions and feelings.
